# Impact of 0.1% sodium hyaluronate and 0.2% sodium hyaluronate artificial tears on postoperative discomfort following cataract extraction surgery: a comparative study

**DOI:** 10.1186/s40662-019-0131-8

**Published:** 2019-02-11

**Authors:** Panagiota Ntonti, Eirini-Kanella Panagiotopoulou, Georgios Karastatiras, Nektarios Breyannis, Sevasti Tsironi, Georgios Labiris

**Affiliations:** 1 Department of Ophthalmology, University Hospital of Alexandroupolis, 68100 Dragana, Alexandroupolis, Greece; 2 Department of Ophthalmology, Papanikolaou General Hospital, Thessaloniki, Greece; 3 Department of Ophthalmology, Naval Hospital of Athens, Athens, Greece; 4 Department of Ophthalmology, Athinaiki Kliniki, Athens, Greece

**Keywords:** Cataract, Postoperative regime, Artificial tears, Surface discomfort index, Hylocomod, Hylogel, Sodium hyaluronate

## Abstract

**Background:**

Recent artificial tear preparations have provided 0.2% concentration of sodium hyaluronate. However, no published data exist on their potential superiority against 0.1% in alleviating dry-eye-disease symptoms in cataract extraction surgery.

**Methods:**

A total of 180 patients that underwent cataract extraction surgery were randomly divided into 2 groups according to their postoperative regime: Study group (SG) received fixed combination of tobramycin and dexamethasone (FCTD) quid for 3 weeks, and additionally 0.2% sodium hyaluronate provided in the COMOD® device quid for 6 weeks. Control group (CG) received fixed combination of tobramycin and dexamethasone (FCTD) quid for 3 weeks, and additionally 0.1% sodium hyaluronate provided in the COMOD® device quid for 6 weeks. The following indexes were evaluated at 3 postoperative checkpoints: 1) Surface discomfort index (SDI) which was derived by four direct 10-scale Likert-type questions that were addressed to the patient and pertained to: a) foreign body sensation (FBS), b) blinking discomfort (BD), c) stinging sensation (SS), d) tearing sensation (TS), 2) Tear break-up time (TBUT), 3) Schirmer’s test, 4) Central corneal thickness (CCT), and 4) Central Corneal Sensitivity (CCS).

**Results:**

Both groups showed reduced CCS values at all postoperative examination points; however, SG participants had significantly better CCS (all *p* < 0.05). SG had better TBUT than CG at the 3rd (*p* = 0.03) and 6th examination points (*p* = 0.04). Moreover, SG had better SDI scores at the 3rd (SDI = 9.26 ± 0.55) and 6th weeks (SDI = 9.47 ± 0.48) vs. CG participants (*p* = 0.03 and *p* < 0.01, respectively).

**Conclusion:**

The increased 0.2% sodium hyaluronate concentration in the artificial tears provided in the COMOD® device seems to address dry-eye-disease symptoms better in patients who underwent phacoemulsification surgery than the 0.1% concentration.

**Trial registration:**

**ClinicalTrials.gov** **Identifier:** NCT03705949 Oct 15, 2018, retrospectively registered.

## Background

Cataract is one of the most prevalent medical conditions, being responsible for about 33% of visual incapacity worldwide and 51% of blindness [1–3]. No reliable conventional treatment means (i.e., drops) exist for cataract, therefore, the treatment of choice is exclusively surgical. Cataract extraction surgery is a minimally invasive technique, usually performed in an outpatient basis [4]. In the vast majority of cases, patients experience a short, uneventful recovery period [5]. Following an uncomplicated operation, the patient has to be administered drops that minimize surgical-induced inflammation and facilitate visual recovery. Regardless of the specific active ingredients, the postoperative regime primarily aims to prevent macular edema, corneal edema, and endophthalmitis [4, 6, 7].

Further to the aforementioned rare but potentially sight-threatening complications, a significant percentage of patients experience symptoms compatible to ocular surface disease (OSD) [8–10]. Among the most frequent OSD symptoms are the foreign body sensation, itching and ocular pain [11, 12]. The pathomechanism of OSD in cataract surgery is associated with both nerve transection during corneal incisions and the local inflammation that contribute to tear film instability [13, 14].

A recent publication from our group indicated the necessity of adding artificial tears to the standard postoperative regime to prevent OSD [15]. According to our report, as well as other similar publications, patients who additionally receive artificial tears in the postoperative regime have significant better scores in break-up-time and significantly less subjective discomfort [15, 16].

Among the prevalent artificial tear medications that have been suggested to alleviate OSD symptomatology in post cataract patients is the 0.1% sodium hyaluronate provided in the continuous monodose system COMOD® (Hylocomod, Farmex, Greece). The COMOD device is an integral airless application system, which enables delivery of multiple sterile doses of a liquid medicinal product. However, 0.2% sodium hyaluronate (Hylogel, Farmex, Greece) that is provided in the exact same COMOD system has been introduced to the market that promises enhanced efficacy due to the double sodium hyaluronate concentration [15]. To our knowledge, no published reports have compared the necessity of 0.2% sodium hyaluronate over 0.1% in cataract extraction surgery. Within this context, this study objective was to compare the impact of these artificial tear preparations on postoperative discomfort following cataract extraction surgery.

## Material/methods

### Setting

This was a prospective, multicentered, randomized trial. Study protocol adhered to the tenets of the Declaration of Helsinki and written informed consent was obtained from all participants. The institutional review board of the Democritus University of Thrace approved the protocol and the study was conducted at the University Hospital of Alexandroupolis, Naval Hospital in Athens, Papanikolaou General Hospital in Thessaloniki, and Athinaiki General Clinic in Athens, Greece between September 2017 and September 2018.

### Participants

Participants were recruited from the Cataract Service of the aforementioned hospitals in a consecutive-if-eligible basis. Eligibility criteria included diagnosis of senile cataract with stage 2 or 3 nuclear opalescence according to the Lens Opacities Classification System III (LOCS-3) grading scale. Exclusion criteria for all study groups included: diagnosis or evidence of dry-eye-disease (DED), IOP-lowering medications, former incisional surgery, former diagnosis of corneal disease, diabetes, autoimmune or mental diseases. By means of a custom computer randomization program, all participants were randomly assigned to two study groups according to the postoperative regime that was prescribed: a) Study group (SG) received fixed combination of tobramycin and dexamethasone (FCTD), (Tobradex, Alcon, Greece) quid for 3 weeks and 0.2% sodium hyaluronate (Hylogel) quid for 6 weeks, and b) Control Group (CG) received Tobradex quid for 3 weeks and 0.1% sodium hyaluronate (Hylocomod) quid for 6 weeks.

### Surgical technique

Despite the fact that four different surgeons performed the operations in the different centers, a consistent surgical methodology was followed to minimize the impact of surgical technique in the study outcomes. For example, all surgeons performed a 2.2 mm, superior-temporal or superior-nasal (eleven o’clock), self-sealing, clear-cornea incision, and two contralateral stabs. They used the same viscoelastic devices and intraocular lenses, exactly as described in a former report from our group [15].

### Data collection

The following parameters were comparatively evaluated one, three and six weeks postoperatively: 1) Surface discomfort index (SDI), 2) Schirmer test, 3) Tear break-up time (TBUT), 4) Central corneal thickness (CCT) using anterior segment optical coherence tomography, and 5) Central Corneal Sensitivity (CCS) using the Cochet-Bonnet aesthesiometer. SDI is a novel corneal discomfort parameter that evaluates: a) foreign body sensation (FBS), b) blinking discomfort (BD), c) stinging sensation (SS), and d) tearing sensation (TS). For detailed presentation of SDI and its validation process please refer to a former publication from our group [15].

### Statistical analysis

An a priori power analysis was performed. For an effect size of 0.35 of the SDI, 72 participants would be required in each group for the study to have a power of 0.8 at the significance level of 0.05. The normality of measured data was evaluated by the Kolmogorov-Smirnov test. Normal distribution data were assessed by Student’s t-test. Non-parametric data were assessed with Mann–Whitney U test. Values at the *p* < 0.05 were considered statistically significant. All statistical analyses were performed with the Medcalc version 9.6.2.0 (Medcalc Software, Mariakerke, Belgium).

## Results

One hundred eighty patients (82 men and 98 women, mean age 72.7 ± 8.28 years) were recruited and randomly assigned study (90 participants) or control (90 participants) groups. Detailed demographic and clinical parameters are presented in Tables 1 and 2. Non-significant differences were detected with respect to age (*p* = 0.69) and BSCVA (*p* = 0.48) among the groups. No parameter demonstrated significant differences between the two groups preoperatively.

**Table 1:** Preoperative data for all participants

Group	No.	Age		BSCVA	
		Years	SD	LogMAR	SD
CG	90	72.9	7.8	0.92	0.07
SG	90	72.4	8.7	0.80	0.05
*p*-value		0.69		0.48	

*CG* control group, *SG* study group, *BSCVA* best-spectacles corrected visual acuity, *SD* standard deviation

**Table 2:** Group comparisons preoperatively

	Preoperative		
Parameter (mean ± SD)	CG	SG	*p*-value
CCT (μm)	537 ± 36	544 ± 34	0.22
CCS (cm)	5.76 ± 1.39	5.74 ± 1.34	0.35
TBUT (secs)	11.51 ± 7.08	11.92 ± 6.92	0.69
Schirmer (mm)	11.64 ± 3.43	11.93 ± 3.60	0.58
FBS	NA	NA	NA
BD	NA	NA	NA
SS	NA	NA	NA
TS	NA	NA	NA
SDI	NA	NA	NA

*CG* control group, *SG* study group, *CCT* central corneal thickness, *CCS* central corneal sensitivity, *TBUT* tear break up time, *FBS* foreign body sensation, *BD* blinking discomfort, *SS* stinging sensation, *TS* tearing sensation, *SDI* surface discomfort index, *SD* standard deviation, *NA* not applicable

**Fig. 1:**
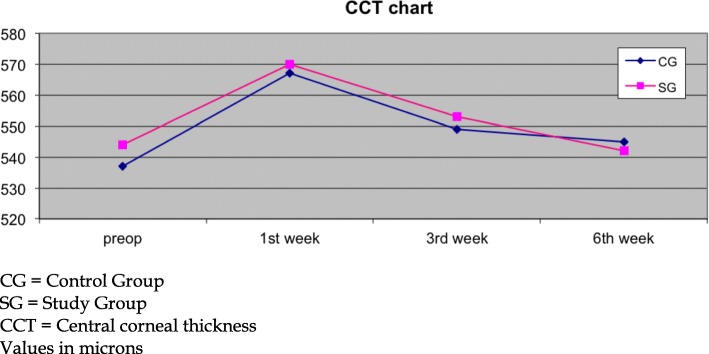
Central corneal thickness

All postoperative comparisons are presented in Tables 3, 4 and 5. While 0.1% sodium hyaluronate participants demonstrated significantly increased CCT values at all examination points in comparison to the preoperative values (all *p* < 0.05), 0.2% sodium hyaluronate participants regained CCT preoperative values at the last examination point (preoperative: 544 ± 34 μm, 6th week: 542 ± 35 μm, *p* = 0.08; Fig. 1).

**Table 3:** Group comparisons (1st postoperative week)

	1st week		
Parameter (mean ± SD)	CG	SG	*p*-value
CCT (μm)	567 ± 43♭	570 ± 44♭	0.66
CCS (cm)	3.34 ± 1.21♭	3.98 ± 1.15♭	0.01*
TBUT (secs)	11.00 ± 6.76	12.04 ± 7.82	0.34
Schirmer (mm)	11.93 ± 3.49	11.47 ± 3.66	0.32
FBS	8.98 ± 0.89	9.01 ± 1.08	0.82
BD	8.92 ± 1.04	9.03 ± 0.84	0.43
SS	8.94 ± 1.03	9.20 ± 0.93	0.08
TS	8.90 ± 0.85	8.98 ± 0.94	0.56
SDI	8.93 ± 0.71	9.06 ± 0.72	0.25

*CG* control group, *SG* study group, *CCT* central corneal thickness, *CCS* central corneal sensitivity, *TBUT* tear break up time, *FBS* foreign body sensation, *BD* blinking discomfort, *SS* stinging sensation, *TS* tearing sensation, *SDI* surface discomfort index, *SD* standard deviation

♭ indicates significant difference with preoperative values

*p < 0.05

**Table 4:** Group comparisons (3rd week postoperatively)

	3rd week		
Parameter (mean ± SD)	CG	SG	*p*-value
CCT (μm)	549 ± 35♭	553 ± 39♭	0.53
CCS (cm)	4.02 ± 1.01♭	4.54 ± 1.22♭	0.04*
TBUT (secs)	12.46 ± 8.07♭	14.68 ± 8.73♭	0.03*
Schirmer (mm)	12.57 ± 3.52♭	12.76 ± 3.42♭	0.72
FBS	9.08 ± 0.82	9.26 ± 0.92	0.17
BD	9.10 ± 0.96	9.26 ± 0.63	0.20
SS	9.12 ± 0.89	9.40 ± 0.73	0.02*
TS	9.02 ± 0.72	9.14 ± 1.11	0.38
SDI	9.08 ± 0.60	9.26 ± 0.55	0.03*

*CG* control group, *SG* study group, *CCT* central corneal thickness, *CCS* central corneal sensitivity, *TBUT* tear break up time, *FBS* foreign body sensation, *BD* blinking discomfort, *SS* stinging sensation, *T* tearing sensation, *SDI* surface discomfort index, *SD* standard deviation

♭ indicates significant difference with preoperative values

*p < 0.05

**Table 5:** Group comparisons (6th week postoperatively)

	6th week		
Parameter (mean ± SD)	CG	SG	*p*-value
CCT (μm)	545 ± 32♭	542 ± 35	0.87
CCS (cm)	4.02 ± 1.04♭	4.56 ± 1.16♭	0.01*
TBUT (secs)	12.52 ± 8.74♭	14.62 ± 9.73♭	0.04*
Schirmer (mm)	13.54 ± 3.90♭	14.21 ± 3.61♭	0.24
FBS	9.12 ± 0.67	9.46 ± 0.85	0.04*
BD	9.34 ± 0.75	9.50 ± 0.57	0.12
SS	9.14 ± 0.89	9.57 ± 0.69	< 0.01
TS	9.18 ± 0.74	9.36 ± 0.69	0.10
SDI	9.27 ± 0.52	9.47 ± 0.48	< 0.01

*CG* control group, *SG* study group, *CCT* central corneal thickness, *CCS* central corneal sensitivity, *TBUT* tear break up time, *FBS* foreign body sensation, *BD* blinking discomfort, *SS* stinging sensation, *TS* tearing sensation, *SDI* surface discomfort index, *SD* standard deviation

♭ indicates significant difference with preoperative values

*p < 0.05

**Fig. 2:**
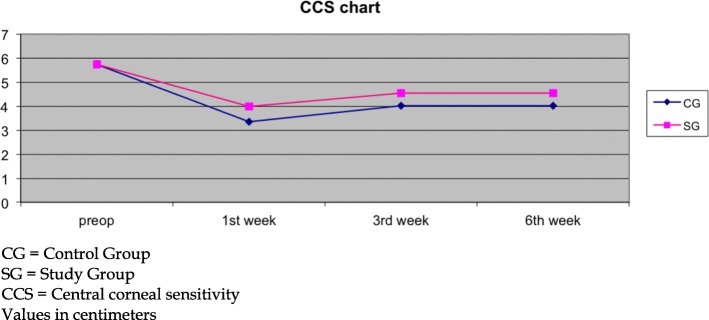
Central corneal sensitivity

With respect to CCS, participants in both groups demonstrated reduced corneal sensitivity at all postoperative examination points. However, despite the overall reduction in CCS, 0.2% sodium hyaluronate members presented significantly better corneal sensitivity vs. control (1st week: SG: 3.98 ± 1.15, CG: 3.34 ± 1.21, *p* = 0.01, 3rd week: SG: 4.54 ± 1.22, CG: 4.02 ± 1.01, *p* = 0.04, 6th week: SG: 4.56 ± 1.16, CG: 4.02 ± 1.04, p = 0.01; Fig. 2).

**Fig. 3:**
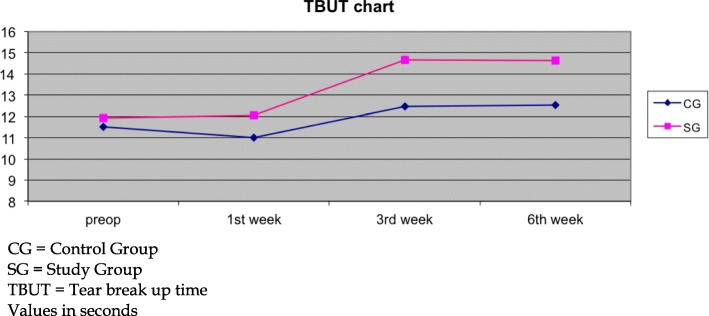
Tear break up time

Significant differences among groups’ participants were detected in the TBUT parameter, as well. Both groups demonstrated non-significant improvement in TBUT at the first postoperative timepoint (both *p* > 0.05). At the 3rd and 6th weeks both 0.1 and 0.2% sodium hyaluronate participants presented significant improvement in comparison to their preoperative values; however, TBUT significantly improved in 0.2% participants in comparison to the 0.1% group (3rd week: SG: 14.68 ± 8.73 s, CG: 12.46 ± 8.07 s, *p* = 0.03, 6th week: SG: 14.68 ± 8.73 s, CG: 12.52 ± 8.74 s, *p* = 0.04; Fig. 3).

**Fig. 4:**
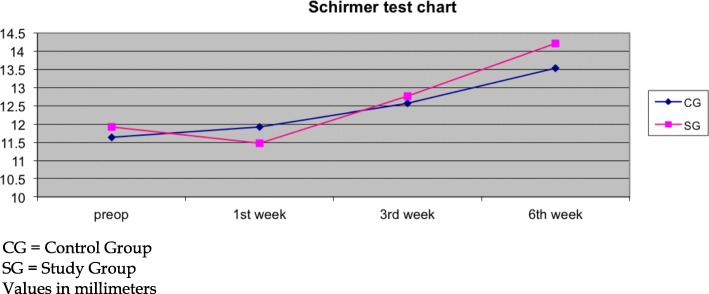
Schirmer’s chart

On the other hand, both groups presented similar results in Schirmer’s test. Significant increase in the Schirmer test was detected at the 3rd and 6th weeks. Non-significant differences could be detected between groups at all timepoints (Fig. 4).

**Fig. 5:**
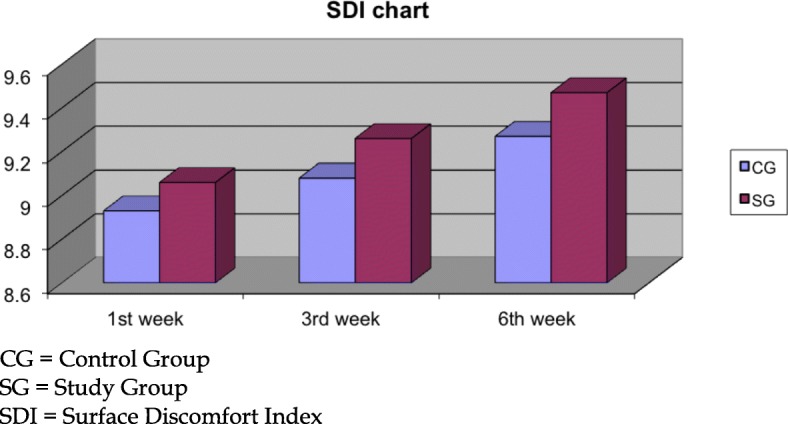
Surface discomfort index

**Fig. 6:**
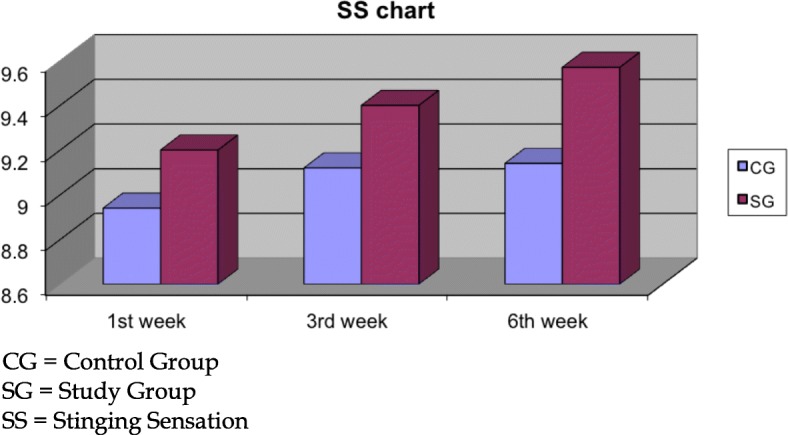
Stinging sensation

**Fig. 7:**
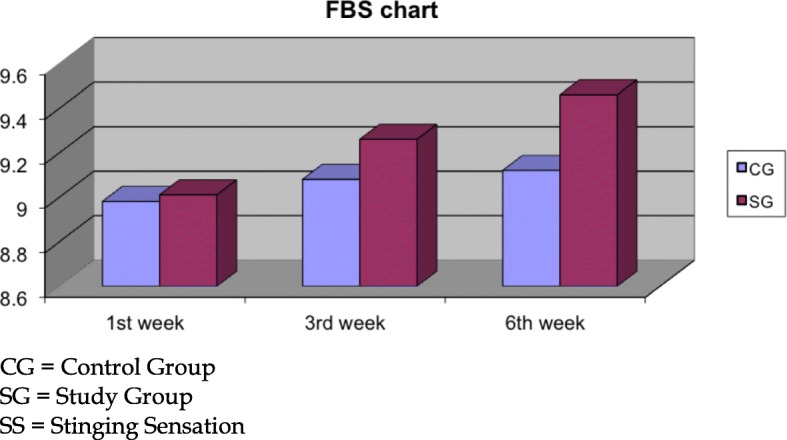
Foreign body sensation

Regarding the subjective discomfort as expressed by our study participants, 0.2% sodium hyaluronate participants demonstrated significant better SDI scores at the 3rd (SG: 9.26 ± 0.55, CG: 9.08 ± 0.60, *p* = 0.03) and 6th (SG: 9.47 ± 0.48, CG: 9.27 ± 0.52, *p* < 0.01) weeks (Fig. 5). The overall improved score in the SDI parameter was primarily attributed to the significant better score in the Stinging Sensation index at the 3rd (*p* = 0.02) and 6th weeks (*p* < 0.01, Fig. 6) and additionally in the Foreign Body Sensation Index at the 6th week (*p* = 0.04, Fig. 7).

## Discussion

Phacoemulsification is considered the most prevalent surgical procedure in ophthalmology both in developed and in developing countries [17, 18]. Despite the advances in cataract-extraction technology, this common surgical technique has been associated with a series of intraoperative and postoperative adverse-effects and complications. Transient corneal edema and reduced corneal sensitivity are two of the mild adverse-effects. Among the more severe ones is the permanent corneal decompensation due to endothelial cell damage. Nevertheless, according to the majority of recently published reports, cataract surgery delivers favorable outcomes in 99% of the cases since patients present impressive visual rehabilitation [10] and high levels of satisfaction [19]. The impressive visual capacity following cataract extraction surgery is not only associated with the removal of the opaque crystalline lens and its replacement with an artificial intraocular lens (IOL), but also due to the integrated properties and characteristics of the IOLs which attempt to address the majority of pre-existing refractive errors and aberrations. It is no surprise that modern cataract-extraction surgery has become part of refractive surgery [20].

Within this context, corneal surface and tear-film have become of major importance to cataract surgeons as it was traditionally for refractive surgeons. However, published experience suggests that the majority of post-cataract patients experience DED-like symptoms that vary in severity and duration [11, 21–25]. Among them are foreign body sensation, burning, stinging sensation, itchiness, tearing, blinking discomfort and pain. Our previous report presented the validation process of a novel surface discomfort index (SDI), which quantified, in a scale from 1 to 10 (best), the overall subjective discomfort feeling that post-cataract patients experience. SDI was constructed by four of the most commonlyexperienced symptoms which acted as components to the parameter (foreign body sensation, blinking discomfort, stinging sensation, and tearing sensation). Moreover, our previous report confirmed former studies and suggested that an artificial tear preparation should be added to the postoperative regime since it alleviates DED-like symptomatology. In fact, 0.1% sodium hyaluronate was found to be equally efficient in alleviating post-cataract DED as the more advanced polyethylene glycol 400/propylene glycol/hydroxypropyl-guar; patients who received aforementioned medications presented significantly less postoperative discomfort than those who received no artificial tears [15].

On the other hand, recent artificial tears preparations doubled the concentration of sodium hyaluronate to 0.2%. However, no comparative clinical trials have been published to confirm a potential additional beneficial impact on DED due to the increased sodium hyaluronate concentration over the standard 0.1% one. Exploring the potential superiority of 0.2% sodium hyaluronate over 0.1% in patients who underwent phacoemulsification surgery was the primary objective of our study.

Regarding sodium hyaluronate, we do know that its viscoelastic properties facilitate the prolonged adhesion of the tear film layer [26]. Moreover, it has excellent moisturization properties and increases the total thickness of the lacrimal film [27]. On the other hand, it also mimics the rheological properties of the aqueous tear layer resulting in its stabilization [28, 29]. In vitro reports demonstrated its antioxidant properties, which minimize the oxidative stress due to the intraoperative procedure and due to the preservatives and active ingredients of the standard postoperative medication [30]. In fact, sodium hyaluronate stimulates ocular surface tissue healing by humidifying the surface of the eye and restores the integrity of the corneal and conjunctival epithelium [31]. Recent reports indicated that sodium hyaluronate is equally efficient to 0.05% cyclosporine in patients with dry eye disease [32].

Although no comparative clinical trials have been published regarding the potential superiority of 0.2% sodium hyaluronate over 0.1% in patients who underwent phacoemulsification surgery, published literature has examined the impact of 0.2 and 0.18% sodium hyaluronate in patients with moderate to severe dry eye with keratitis or keratoconjunctivitis. Nonsuperiority of 0.2% over 0.1% sodium hyaluronate was demonstrated for the reduction of ocular surface lesions. However, some parameters, such as staining score and adverse effects, presented better scores in 0.2% sodium hyaluronate concentrations [33, 34]. Of note, even higher concentrations of sodium hyaluronate (0.3%) was found to perform better in laboratory and clinical settings [35, 36].

Our study outcomes indicated a potential overall superiority of the 0.2% concentration over the 0.1% in the majority of studied parameters: a) SG participants regained their preoperative CCT values at the last examination point, b) although both groups demonstrated significant reduced CCS values at all examination points, patients that received 0.2% sodium hyaluronate had significant better CCS, c) TBUT was significantly better in SG participants than in CG ones at the 3rd and 6th postoperative weeks. Last but not least, the overall surface discomfort that phacoemulsification patients experience was significantly less in 0.2% sodium hyaluronate patients at the 3rd and 6th weeks following their operation. It seems that the increased concentration of sodium hyaluronate has an additional beneficial impact on the stinging sensation and the foreign body sensation, since these were the parameters of SDI that were significantly improved in study group patients.

## Conclusion

A robust feature of our study is the number of participants that populated the study and control groups and the multi-centered design. Among the potential weaknesses is the limited number of parameters that were evaluated (i.e., corneal staining was not evaluated). Nevertheless, our study outcomes suggest that 0.2% sodium hyaluronate artificial tears medication is superior to the 0.1% formulation in patients that underwent cataract extraction surgery, and it should be considered as an important component of the postoperative regime.
